# A Model of Left Ventricular Dysfunction Complicated by CAWS Arteritis in DBA/2 Mice

**DOI:** 10.1155/2012/570297

**Published:** 2012-07-08

**Authors:** Naoto Hirata, Ken-ichi Ishibashi, Tatsuya Usui, Jiro Yoshioka, Satoru Hata, Yoshiyuki Adachi, Noriko Nagi-Miura, Shin Ohta, Naohito Ohno

**Affiliations:** ^1^Department of Pharmacy, Nagano Red Cross Hospital, 5-22-1 Wakasato, Nagano, Nagano 380-8582, Japan; ^2^Laboratory for Immunopharmacology of Microbial Products, School of Pharmacy, Tokyo University of Pharmacy and Life Science, 1432-1 Horinouchi, Hachioji, Tokyo 192-0392, Japan; ^3^Department of Cardiology, Nagano Red Cross Hospital, 5-22-1 Wakasato, Nagano, Nagano 380-8582, Japan; ^4^Department of Pathology, Nagano Red Cross Hospital, 5-22-1 Wakasato, Nagano, Nagano 380-8582, Japan; ^5^Department of Pharmaceutical Health Care and Science, Tokyo University of Pharmacy and Life Science, 1432-1 Horinouchi, Hachioji, Tokyo 192-0392, Japan

## Abstract

It was reported previously that a *Candida albicans* water-soluble fraction (CAWS), including a mannoprotein and **β**-glucan complex, has strong potency in inducing fatal necrotizing arteritis in DBA/2 mice. In this study, histopathological changes and cardiac function were investigated in this system. One mg/day of CAWS was given to DBA/2 mice via peritoneal injection for five days. The CAWS-treated DBA/2 mice were induced aortitis and died at an incidence of 100% within several weeks. Histological findings included stenosis in the left ventricular outflow tract (LVOT) and severe inflammatory changes of the aortic valve with fibrinoid necrosis. Cardiomegaly was observed and heart weight increased 1.62 fold (*P* < 0.01). Echocardiography revealed a severe reduction in contractility and dilatation of the cavity in the left ventricle (LV): LV fractional shortening (LVFS) decreased from 71% to 38% (*P* < 0.01), and the LV end-diastolic diameter (LVDd) increased from 2.21 mm to 3.26 mm (*P* < 0.01). The titer of BNP mRNA increased in the CAWS-treated group. Severe inflammatory changes resulting from CAWS brought about lethal LV dysfunction by aortic valve deformation with LVOT stenosis. This system is proposed as an easy and useful experimental model of heart failure because CAWS arteritis can be induced by CAWS injection alone.

## 1. Introduction

Cardiovascular disease is the major cause of death and one of the largest burdens on healthcare resources worldwide. Clinical guidelines for the treatment of cardiovascular disorders have been developed by the analysis of high quality Evidence-Based Medicine (EBM) methodology. However, it is considered that the existence of a superior animal model is indispensable for the development of new therapeutic agents and methods.

It was reported previously that pathogen-associated molecular patterns (PAMPs) [1] in the *Candida albicans* water-soluble fraction (CAWS) have a strong induction-potency for murine vasculitis and show acute lethal toxicity in certain mouse strains through various biological activities [2–5]. CAWS includes water-soluble mannoprotein and *β*-glucan complex obtained from the culture fluid of *C. albicans *strain NBRC 1385 [6]. On the other hand, CADS, which comprises *C. albicans *cell extract and originates from the stool sample of a Kawasaki disease patient, has been reported to induce murine vasculitis similar to Kawasaki disease coronary arteritis [7, 8]. Furthermore, in an additional study on *Candida*-derived agents, CAWS induced severe vasculitis at a higher rate of incidence than CADS in many mouse strains, and there are reports on this valuable animal model system in research on systemic immune responses and inflammatory vascular disease [3, 5, 9]. In particular, CAWS caused severe necrotizing coronary arteritis and aortitis in DBA/2 mice with an incidence of 100% after intraperitoneal administration, and all the mice died within several weeks [10, 11]. 

The chronic mortality of CAWS arteritis is found only with DBA/2 mice, but CAWS arteritis is not fatal in other mouse strains that are sensitive to CAWS. Among mice that are susceptible to CAWS arteritis, the reason why only DBA/2 mice die is not clearly understood.

In this report, for the purpose of establishing the validity of DBA/2 mice as a heart failure model, the influence of CAWS arteritis on cardiac hypertrophy was investigated for its effect on cardiac function using echocardiography. Furthermore, cardiac function was analyzed by measuring type B natriuretic peptide (BNP) levels, which are used a humoral heart failure marker. From the present findings, this study proposes a simple and easy animal model of left ventricular dysfunction induced by CAWS.

## 2. Materials and Methods

### 2.1. Extraction and Preparation of CAWS

CAWS was prepared in accordance with the previous report [6]. *C. albicans* strain NRBC 1385 was cultured initially in complete synthetic culture medium (C-limiting medium) in acidic conditions at pH 5.2 C-limiting medium which contains sucrose 10 g, (NH_4_)_2_SO_4_ 2 g, KH_2_PO_4_ 2 g, CaCl_2_·2H_2_O 0.05 g, MgSO_4_·7H_2_O 0.05 g, ZnSO_4_·7H_2_O 1 mg, CuSO_4_·5H_2_O 1 mg, FeSO_4_·7H_2_O 0.01 g, and biotin 25 mg (per liter). Culture medium was maintained without pH adjustment while aerating with five litter/min room air at 27°C in a churning incubator at 400 rpm during the culture. After two days incubation, culture medium was mixed with an equal volume of ethanol and left overnight. The ethanol insoluble fraction was collected and water-soluble compounds were isolated by added distilled water to the precipitate. The water-soluble compounds were precipitated by added ethanol again and left overnight. CAWS was obtained from the precipitate dried with acetone. Each lot of CAWS used for this study was checked for the following physicochemical and biochemical parameters: endotoxin content, reactivity with anti-*Candida *monovalent antiserum, *β*-glucan content, elemental analysis for carbon, hydrogen, and nitrogen content, and acute fatal activity by the previous report had the same properties as the lots used in the present study.

### 2.2. Maintenance of Mice, Survival, and Histopathological Observation

The handling and treatment of laboratory animals conformed to the Tokyo University of Pharmacy and Life Science laboratory animal handling code. DBA/2 male mice of four and five weeks of age (provided from Japan SLC Co., Ltd.) were treated by intraperitoneal administration of CAWS at a rate of one mg/mouse for consecutive five days. Echocardiography was performed on a test bed maintained at a moderately warm temperature. After CAWS administration, mice were sacrificed every week by intravenous injection of KCl. Their organs were collected and sliced into serial sections for pathological observation using an optical or stereoscopic microscope. The pathological changes of the heart and vascular tissues slices were assessed by staining with Hematoxylin and Eosin (HE) stain. Tissue samples for electron microscopy were collected two weeks after CAWS injection and the images of transmission electron microscope (JEOL JEM-2000EX) were provided by the technical supplier (Hanaichi Co., Ltd.).

### 2.3. Echocardiography

A ProSound SSD-6500SV (ALOKA Co., Ltd.) linear transducer and neonatal probe were used for the determination of murine cardiac function. Mice were anesthetized using Nembutal 40 mg/kg by intraperitoneal administration, and ultrasonic permeable materials were sandwiched between a mouse and the probe to restrain the animal. Echocardiography was performed on a test bed maintained at a moderately warm temperature.

### 2.4. Extraction of Total RNA and Reverse Transcription-Polymerase Chain Reaction (RT-PCR)

Total RNA was obtained isolating a fixed protocol of chloroform extraction from myocardial tissue after homogenizing at low temperature (less than 4°C) in ISOGEN (Nippon GENE Co., Ltd.). The total RNA concentration and purity were measured using a NanoDrop ND-1000 UV Spectrophotometer (NanoDrop Technologies, Inc.). The reaction mixture was prepared from an RT-PCR Kit (Takara Bio Co., Ltd.) as follows for template RNA degeneration and the reverse transcription reaction. The PCR primers used for murine *β*-actin were sense 5′-GCCATGGATGACGATATCGCT-3′ and antisense 5′-TCATGAGGTAGTCTGTCAGGT-3′ (product size 574 bp), and for natriuretic peptide type B (BNP) sense 5′-ATGGATCTCCTGAAGGTGCTG-3′ and antisense 5′-GTGCTGCCTTGAGACCGAA-3′ (product size 241 bp) [12, 13]. The PCR reaction products were isolated by electrophoresis on a 1% agarose gel. The 574 bp band of *β*-actin and the 241 bp band of BNP were detected by ethidium bromide staining and UV irradiation. The band patterns were recorded digitally using a fluorescence intensimeter (IR LAS-1000, Fuji Film Co., Ltd.).

### 2.5. Statistical Analysis

Data are presented as mean ± SEM. The survival data were calculated by the Kaplan-Meier method and compared using Fisher's exact test between the untreated and CAWS treatment groups for the survival ratio at each time point. Parameters determined by echocardiography and of heart weight were examined using Student's *t*-test between untreated and CAWS treatment groups. A value of *P* < 0.05 was considered to be statistically significant. N.D. indicates no statistical difference.

## 3. Results

### 3.1. Survival of CAWS Treatment DBA/2 Mice

**Figure 1:**
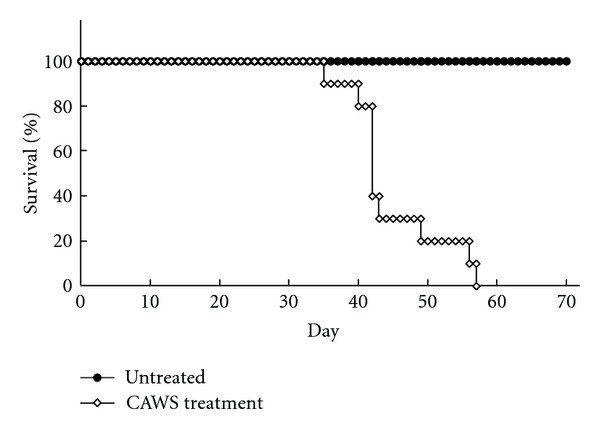
Kaplan-Meier analysis of CAWS arteritis in DBA/2 mice. CAWS treatment DBA/2 mice were administered with CAWS one mg/mouse via peritoneal injections for five consecutive days. DBA/2 mice at four or five weeks of age were maintained in specific pathogen free (SPF) conditions and observed every day. All CAWS treatment mice died between five and eight weeks after treatment. *P* < 0.005 for survival of untreated versus CAWS treatment mice at six weeks after the CAWS treatment; *n* = 8 (control) and *n* = 10 (CAWS treatment).

DBA/2 mice at four or five weeks of age were administered with CAWS at one mg/mouse via the peritoneum for five consecutive days and maintained in specific pathogen free (SPF) conditions. All CAWS treatment DBA/2 mice died between five and eight weeks after CAWS administration (Figure 1). One mouse that died after a sudden convulsive seizure was found in the CAWS treatment group during the observation period.

### 3.2. Development and Progress of the CAWS Arteritis

**Figure 2:**
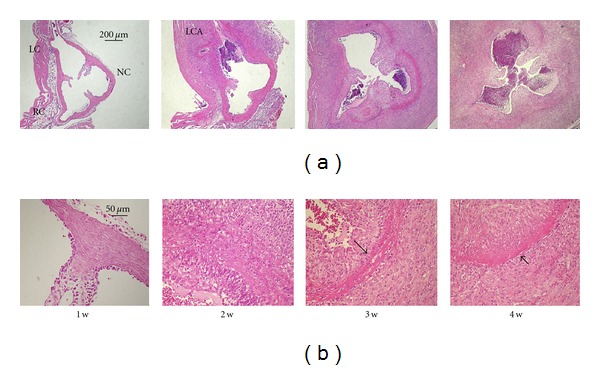
CAWS arteritis development and progress in DBA/2 mice. Cardiovascular tissue was sampled every week after CAWS administration, and induced aortitis was observed with an incidence of 100%. The first week panel (a, b—1w) shows slight aortic valve thickening and endocardial inflammation of the Valsalva sinuses; left coronary (LC) sinus, right coronary (RC) sinus, and noncoronary (NC) sinus. The second week panel (a, b—2w) shows expansion of the severe inflammatory lesion around the left coronary arteries (LCA). The third and fourth week panels (a, b—3w and 4w) show the features of the necrosis-related vasculitis. Inflammatory cells have spread to all the layers and sinuses with fibrinoid necrosis in the elastic lamina (arrows). Severe stenosis was found in coronary arteries because of inflammatory cells accumulation.

**Figure 3:**
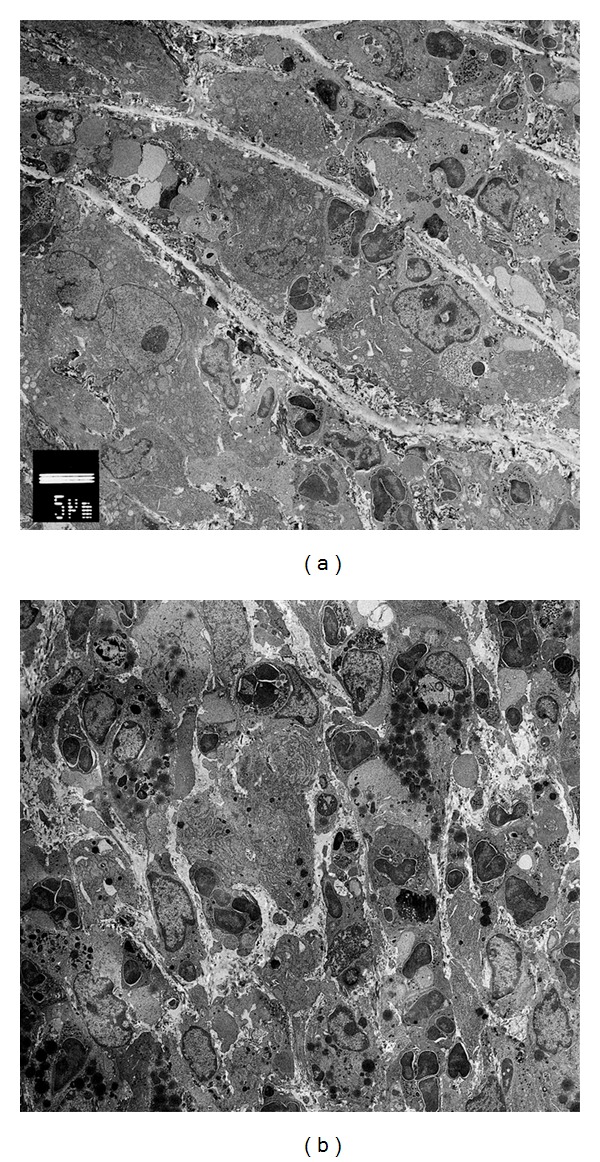
The tissue image of the CAWS arteritis by electron microscope. The images demonstrated direct invasion to the elastic fiber (a) and the activated inflammatory cells accumulation in the abscess-like lesion (b).

**Figure 4:**
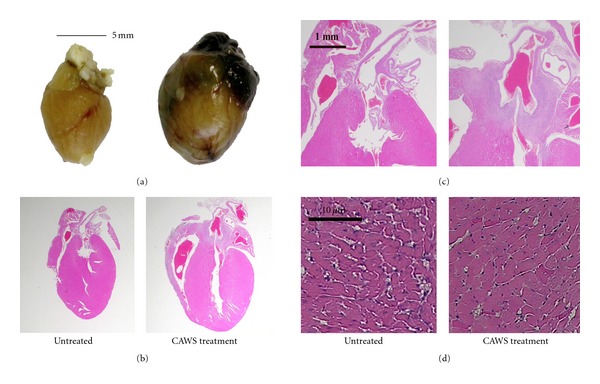
Cardiomegaly and histopathological changes of the aortic root due to CAWS arteritis. Macroscopic cardiomegaly and concentric left ventricular hypertrophy were observed in the CAWS treatment group (a, b). The panels (c) represent enlarged views of each aortic valve. Inflammatory cells were located mainly in the aortic valve and Valsalva sinus, and severe stenosis of the left ventricular outflow tract (LVOT) was found in CAWS arteritis. There was no evidence of necrosis or fibrosis to indicate broad infarctions in the left ventricular wall. The cross sectional area of CAWS-treated cardiomyocyte was increased by 1.14 fold (d).

The development and progress of CAWS arteritis took between three and four weeks. Initially, inflammatory cells invaded into the left coronary (LC) and right coronary (RC) sinus of Valsalva, and then the cells were delayed and invaded the noncoronary (NC) sinus (Figure 2—1w and 2w). These severe inflammatory lesions localized to the proximal coronary arteries and the Valsalva sinus including the aortic valve (Figure 2—2w). Inflammatory cells mainly including neutrophils invaded from the adventitia side to the media gradually, and cell accumulation progressed in the intima synchronously (Figure 2—2w and 3w). After the third week of CAWS administration, the inflammatory lesion spread to all layers of the aortic tissues and Valsalva sinuses with fibrinoid necrosis of the media (Figure 2—3w and 4w). The tissue image by the electron microscope demonstrated the inflammatory cells broke through the elastic fiber and infiltrated into the media side of the intima (Figure 3(a)), and various inflammatory cells accumulated, but these were not classified in detail (Figure 3(b)).

### 3.3. Anatomical and Histopathological Features of CAWS Treatment Hearts

**Figure 5:**
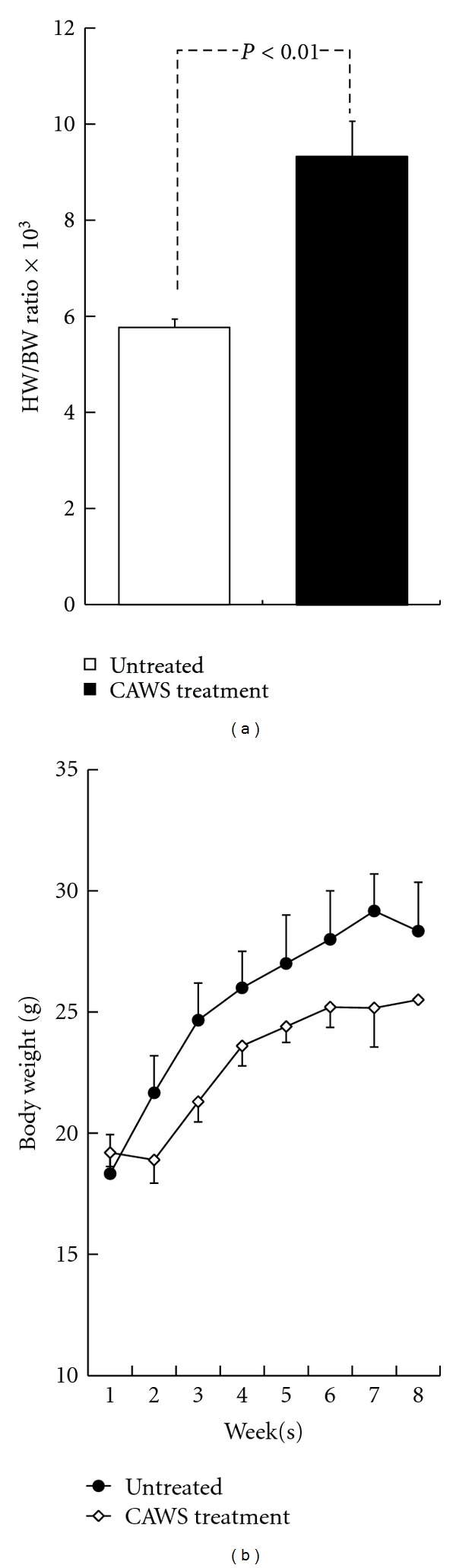
Heart weight and time course of body weight in CAWS arteritis. (a) The column graph represents the heart weight (HW)/body weight (BW) ratio, which increased significantly by 1.62-fold (*P* < 0.01); *n* = 8 (untreated) and *n* = 7 (CAWS treatment). (b) The time course of body weight with/without CAWS treatment DBA/2 mice.

Significant increases in heart weight and cardiomegaly were observed in the CAWS treatment group at six weeks after CAWS administration (Figures 4(a) and 4(b), Figure 5(a)). On the other hand, the body weight of the CAWS treatment group tended to be about 10% lower than the untreated group (Figure 5(b)). A severe accumulation of inflammatory cells like an abscess was found in the Valsalva sinus. The left ventricular outflow tract (LVOT) stenosis and aortic valve deformation were found (Figure 4(c)). Scars of myocardial infarction were observed in the right ventricle wall, but abnormal changes were not found in other valves and there was no evidence of infarction in the left ventricular myocardial tissues. The cross sectional area of CAWS-treated cardiomyocyte was increased though there was no significant difference (Figure 4(d)).

### 3.4. Cardiac Function of CAWS Arteritis Detected by Echocardiography and BNP Induction

**Figure 6:**
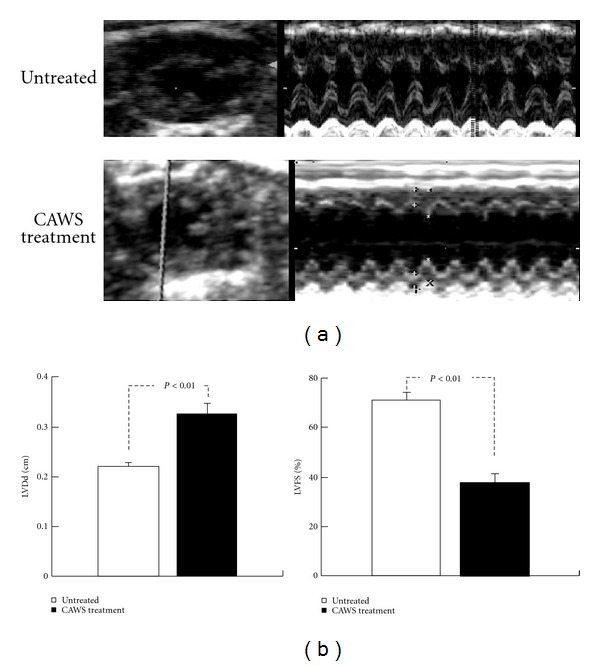
Comparison of the cardiac function measured by echocardiography in untreated and CAWS treatment DBA/2 mice. CAWS treatment DBA/2 mice developed left ventricular dilution and dysfunction between six and seven week after CAWS administration (a). Echocardiography revealed a diffuse, severe reduction of contractility in the left ventricle after CAWS treatment. Left ventricular fractional shortening (LVFS) decreased from 71% to 38% (*P* < 0.01), and dilation of the left ventricular diastolic dimension (LVDd) was observed from 2.21 mm to 3.26 mm (*P* < 0.01) (b); *n* = 10 (untreated) and *n* = 6 (CAWS treatment).

**Figure 7:**
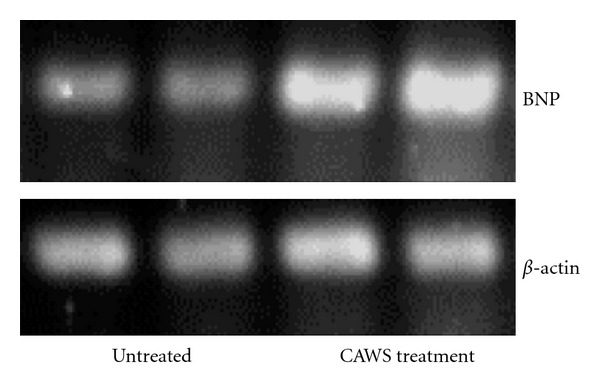
mRNA expression of murine BNP in CAWS arteritis. The cDNA levels of murine BNP were examined by RT-PCR and found to be increased.

Ten DBA/2 mice each in the CAWS treatment and untreated groups were prepared for echocardiography. DBA/2 mice were tested after the sixth week from CAWS administration, but two mice had died in the CAWS treatment group before echocardiographic examination. Mice were anesthetized by intraperitoneal injection with Nembutal 40 mg/kg before the examination. All untreated mice awoke normally after the examination, but two mice in the CAWS treatment group with the severe cardiac dysfunction died because of cardiac arrest during echocardiographic examination (Table 1). The examination found a severe reduction in contractility and dilatation of the cavity in the left ventricle (Figure 6(a)). Dilation of left ventricular end-diastolic dimensions (LVDd) and a decrease in left ventricular fractional shortening (LVFS) were observed in the CAWS treatment group (Figure 6(b)). In this study, the determination of the exact aortic diameter and transmitral flow pattern of left ventricle were difficult to detect by echocardiography. The bloodstream and pressure increase were found in the LVOT and aortic valve, but were not able to show significant difference because only a few results were acquired (data not shown). Furthermore, BNP, which is a marker of heart failure, was measured by the luminous intensity of the band obtained by RT-PCR. The BNP bands showed remarkable enhancement in DBA/2 mice at six weeks after CAWS administration (Figure 7).

## 4. Discussion

The cause of chronic lethal toxicity of CAWS in DBA/2 mice has not been determined sufficiently in previous reports that examined this model only from the viewpoint of arteritis [4, 10, 11]. From previous histopathological reports with high dosage of CAWS, the features of CAWS arteritis were described as necrotizing angiitis, because of the accumulation of various inflammatory cells accompanied by fibrinoid necrosis in the aortic valve and Valsalva sinuses, but other significant abnormalities in CAWS-treated heart were not found [11]. Moreover, severe changes that could be the cause of death due to CAWS were not found in any other main internal organs (digestive system, respiratory system, or cerebral nervous system) previously. It was considered that the weight loss of CAWS-treated group was related to inflammatory cytokines because a lot of inflammatory cytokines production was induced by the CAWS treatment in vitro.

In this histopathological study, inflammatory cells accumulation was located in the aortic root, but there was no evidence of the left ventricular myocarditis or infarction by CAWS treatment. Therefore, the concentric-like left ventricular hypertrophy strongly suggested that the LVOT stenosis and aortic valve deformity brought about left ventricular pressure overload after development of CAWS arteritis in several weeks. The direct measurement of the pressure gradient by using microcatheter was extremely difficult because very practiced technique was needed for operation in the murine aorta of less than one millimeter. By the echocardiography, the bloodstream and pressure increase were found in the LVOT and aortic valve of CAWS-treated mice though there were only a few data. However these data strongly suggested the existence of LVOT stenosis. On the other hand, the results of echocardiography revealed decreasing left ventricular contractility and eccentric-like left ventricular hypertrophy just before death in the CAWS-treated mice. The reasons for this process considered that the left ventricular concentric hypertrophy progressed to dilated phase. In addition, because the two mice caused cardiac arrest during echocardiography test, it was suggested that CAWS-treated mice developed severe cardiac dysfunction of the terminal stage.

The cross-sectional area of CAWS-treated cardiomyocyte was increased, but there was no evidence of fibrosis because only HE stain was performed in this experiment. Furthermore, in the previous report [11], interstitial fibrosis was not found in the limited area around inflammatory lesion with Elastica van Gieson (EVG) stain. Therefore it was considered further data needed to prove fibrosis of the myocardium. BNP was measured as the most sensitive marker of heart failure in clinical settings and secreted by mainly extended ventricular cardiomyocytes [14]. Accordingly, high titer of BNP with cardiomyocyte hypertrophy suggested left ventricular overload by LVOT stenosis and aortic valve disease.

 In experimental coronary artery ligation models and atherosclerosis progression models by apolipoprotein-E deficiency, left ventricular myocardial infarction, and fibrosis were often found [15, 16]. However, left ventricular necrosis and fibrosis to indicate myocardial infarction were not found in CAWS arteritis. Furthermore, echocardiography revealed diffuse wall hypokinesis in the left ventricle, but akinesia in a specific segment of wall was not found. It was suggested that the influence of the ischemia did not cause infarction of the left ventricle despite of the ostial coronary lesion in CAWS arteritis. On the other hand, the myocardial infarction of the right ventricle was found in the CAWS-treated group. DBA/2 mouse is a strain that has severe pericarditis with calcification in wild type. Especially remarkable change is observed in right ventricle pericardium. It is difficult to explain the reason why myocardial infarction was developed only in the right ventricle, but it was suspected that the native severe pericardium calcification and the direct invasion of the inflammatory cells were causes of the necrosis.

In the previous studies, CBA/J mice were found to be resistant to CAWS by immunosuppressive IL-10 induction [17], but significant inflammatory cytokines production and neutrophil activation were observed in sensitive murine strains, such as DBA/2, ICR, C57BL/6, BALBc, C3H/HeN, and C3H/HeJ [3–5, 18]. CAWS-sensitive mice, except for strain DBA/2, did not die despite developing arteritis. One of the reasons for the lethal cardiac toxicity is that DBA/2 mice show the most severe progression of arteritis among CAWS-sensitive murine strains. In addition, wild-type DBA/2 mice were found to have spontaneous extensive calcification of the epicardium and myocarditis of the right ventricle [19]. The origin of the epicardial calcification has been explained as the necrosis and mineralization of subepicardial myocytes, which may indicate epicardial inflammation and dystrophic calcinosis in DBA/2 mice [20]. In the present study it was not possible to determine ventricular dysfunction in wild-type DBA/2 mouse compared with other less-susceptible strains.

In the report of CADS-vasculitis [21], abscess and necrosis were observed in the renal artery, lymph nodes and the liver, but the lesions of CAWS arteritis were localized to the aortic root including ostial coronary arteries and aortic valve.

An antiaortic antibody (autoantibody with collagen reactivity) and antineutrophil cytoplasmic antibody (ANCA) have been suggested to participate in human vasculitis [22]. An anticardiac myosin autoantibody was reported in a Kawasaki disease patient [23], though there seems to be no myocarditis in CAWS arteritis. In this study, it was not possible to prove the existence of autoantibodies or to clarify the reason why severe lesions were limited to proximal cardiovascular tissues, but further studies are proceeding to examine the involvement of autoantibodies.

Recently, there have been many reports suggesting a relationship with microbial infection or antigens and vascular disease. There are reports to suggest the participation of ANCA-associated vasculitis and infection [24]. In addition, research into the mechanisms of innate immunity in response to infectious diseases has advanced rapidly. In particular, immunostimulatory agents of microbial origin, such as the lipopolysaccharide (LPS), have been identified as pathogen-associated molecular patterns (PAMPs) [1]. It has been recognized that pattern recognition receptors (PRRs), including toll-like receptors (TLRs), play an important role in the biological activities of PAMPs [25]. TLR 2 and MyD 88 are related to the murine Kawasaki disease model caused by *Lactobacillus casei* cell wall extract (LCCWE) but no evidences of cooperating with TLR 4 in LCCWE arteritis [26]. Furthermore, there is a report of increased expression of CD180, which is regarded as a B-cell surface TLRs homologue, in viral infection and a Kawasaki disease patient [27]. Although TLR 2 and/or TLR 4 are associated with cardiovascular remodeling, atherosclerosis, and *Candida* derived mannan activity [28, 29], at least TLR 4 seems not to be required in CAWS arteritis because it was induced even in C3H/HeJ mice, which is a TLR 4-mutant strain and shows a weak response to LPS [2, 5]. Lectin receptor is related to the signaling of PAMPs, and Dectin-1 and -2 are known as lectin receptors of *β*-glucan and *α*-mannan, those are representative PAMPs of fungal origin [30, 31]. In a recent study, *Candida *mannan and glucan require the cooperation of lectin and TLRs to induce immune responses [32]. It was speculated that CAWS activates the human complement system strongly through a lectin pathway when lectin receptor participates in the vasculitis initiation activity of CAWS [10]. Therefore, it seems that cooperation with various receptors and immune molecules is involved in the biological activity of CADS and CAWS. Infection with *C. albicans* does not lead to the direct induction of vasculitis, and the activity of CAWS depends on its higher structure. In particular, it is recognized that the structure of mannan is influenced by the culture conditions of *C. albicans* [3, 6, 10].

**Figure 8:**
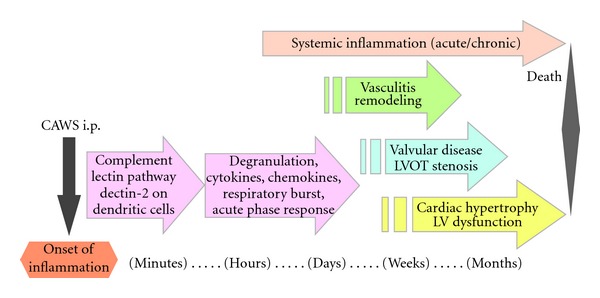
Summarized time course of CAWS-induced systemic inflammation and vasculitis.

In conclusion, these inflammatory changes of CAWS arteritis bring about aortic valvular disease and LVOT stenosis. It was suggested that the progression of CAWS arteritis is classified into the following three stages, the first stage involves the development of vasculitis, the second stage includes complicated myocardial remodeling with hypertrophy by overload, and the last stage is fatal severe left ventricular dysfunction and sudden death (Figure 8). It is proposed that the easy and unique heart failure model induced by CAWS arteritis, without the requirement for specific techniques such as a minute surgical procedure or virus infection, constitutes a valuable model system. Furthermore, large numbers of study animals can be induced to develop angiitis at the same time through simple intraperitoneal injections of CAWS. CAWS arteritis and the complications of fatal heart failure form a simple and useful experimental model of cardiovascular diseases for preclinical investigations.

## Figures and Tables

**Table 1:** Cardiac parameter calculated from echocardiography.

	Untreated (*n* = 10)	CAWS treatment (*n* = 6^∗^)
LVDd (cm)	0.221 ± 0.008	0.326 ± 0.023^†^
LVDs (cm)	0.066 ± 0.009	0.204 ± 0.023^†^
LVEF (%)	96.438 ± 0.860	74.044 ± 5.132^†^
LVFS (%)	70.963 ± 3.760	37.928 ± 4.128^†^
HR (bpm)	317.648 ± 18.628	318.750 ± 18.414^‡^

^∗^Four CAWS treatment mice died before and during examination, ^†^: *P* < 0.01, ^‡^: N.D.

Left ventricle (LV): LVPWd; LV posterior wall thickness in diastole, LV end-diastolic diameter, LVDs; LV end-systolic diameter, LVEF; LV ejection fraction, LVFS; LV fractional shortening, HR; heart rate.
